# Highlighting the interplay of microRNAs from *Leishmania* parasites and infected-host cells

**DOI:** 10.1017/S0031182021001177

**Published:** 2021-10

**Authors:** Sajad Rashidi, Reza Mansouri, Mohammad Ali-Hassanzadeh, Esmaeel Ghani, Afshin Barazesh, Mohammadreza Karimazar, Paul Nguewa, Eugenio Antonio Carrera Silva

**Affiliations:** 1 Department of Parasitology and Mycology, School of Medicine, Shiraz University of Medical Sciences, Shiraz, Iran; 2 Department of Immunology, Faculty of Medicine, Shahid Sadoughi University of Medical Sciences and Health Services, Yazd, Iran; 3 Department of Immunology, School of Medicine, Jiroft University of Medical Sciences, Jiroft, Iran; 4 Endocrinology and Metabolism Research Center, Hormozgan University of Medical Sciences, Bandar Abbas, Iran; 5 Department of Microbiology and Parasitology, Faculty of Medicine, Bushehr University of Medical Sciences, Bushehr, Iran; 6 University of Navarra, ISTUN Instituto de Salud Tropical, Department of Microbiology and Parasitology, IdiSNA (Navarra Institute for Health Research), c/Irunlarrea 1, 31008 Pamplona, Spain; 7 Institute of Experimental Medicine, CONICET-National Academy of Medicine, Buenos Aires, Argentina

**Keywords:** *Leishmania*, Leishmaniasis, miRNAs, miRs, therapeutic targets

## Abstract

*Leishmania* parasites, the causative agents of leishmaniasis, are protozoan parasites with the ability to modify the signalling pathway and cell responses of their infected host cells. These parasite strategies alter the host cell environment and conditions favouring their replication, survival and pathogenesis. Since microRNAs (miRNAs) are able to post-transcriptionally regulate gene expression processes, these biomolecules can exert critical roles in controlling *Leishmania*-host cell interplay. Therefore, the identification of relevant miRNAs differentially expressed in *Leishmania* parasites as well as in infected cells, which affect the host fitness, could be critical to understand the infection biology, pathogenicity and immune response against these parasites. Accordingly, the current review aims to address the differentially expressed miRNAs in both, the parasite and infected host cells and how these biomolecules change cell signalling and host immune responses during infection. A deep understanding of these processes could provide novel guidelines and therapeutic strategies for managing and treating leishmaniasis.

## Graphical abstract

**Figure:**
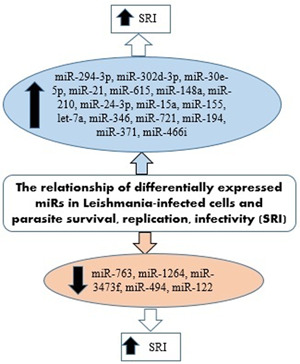

## *Leishmania* parasites

Leishmaniasis is a neglected disease in tropical and subtropical regions caused by the intracellular parasites from the genus *Leishmania* and transmitted by bites of infected sand fly vectors (Torres-Guerrero *et al*., 2017). Cutaneous leishmaniasis (CL), mucocutaneous leishmaniasis (MCL) and visceral leishmaniasis (VL) are three important forms of this disease (Torres-Guerrero *et al*., 2017). Metacyclic promastigotes, the infective forms of these parasites after the bite, are phagocytosed by the host macrophages, turning later into amastigotes to proliferate inside these cells and cause progressive infection (Frank *et al*., 2015; Rashidi *et al*., 2018). The host immune cells activate macrophage killing programme to eliminate the intruder, however the ability of *Leishmania* parasite to evade or suppress the host immune response positively correlates with infection progression (Gupta *et al*., 2013). Although the chemotherapy is considered the most effective way to treat leishmaniasis, due to the presence of antimonial drug resistance and side effects of such compounds, there is an increased need of novel therapeutic targets and new fully effective drugs available for treatment (Pérez-Victoria *et al*., 2011; Rashidi *et al*., 2020*b*, 2021). Identifying the parasite strategies to alter the macrophage defence mechanisms and to survive within these cells, could bring new insights and suggest novel therapeutic targets for leishmaniasis (Rabhi *et al*., 2012; Rashidi *et al*., 2020*a*; Kalantar *et al*., 2021). Accordingly, since microRNAs (miRNAs, miRs) are involved in most of the mechanisms relevant to the parasite pathogenicity and survival in the infected host cells, their inhibition could be a new therapeutic approach to control parasite proliferation and immune evasion (Hashemi *et al*., 2018*a*).

## miRNAs

**Fig. 1.**
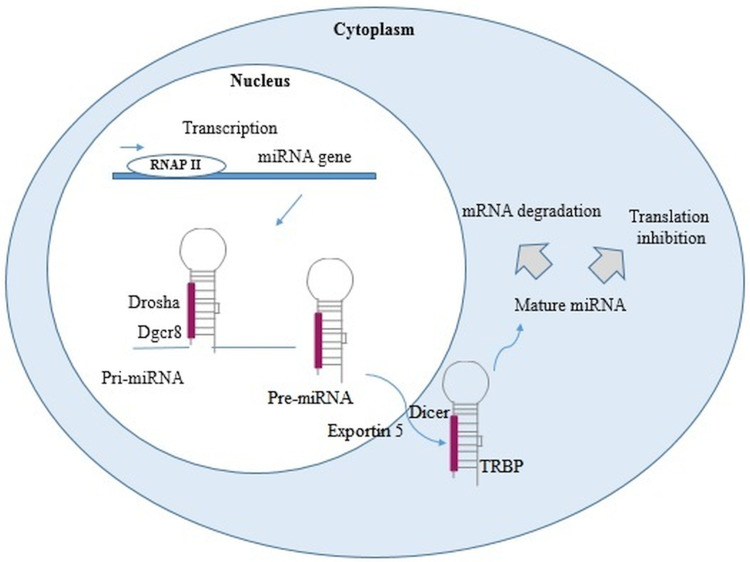
miRNA biogenesis. In the nucleus, RNA polymerase II or III transcribed miRNA genes into primary miRNAs (pri-miRNAs). Pri-miRNAs, after being processed by Drosha and DiGeorge syndrome Critical Region 8 (DGCR8), become into miRNAs precursor (pre-miRNAs). These pre-miRNAs are exported into the cytoplasm by exportin 5 and Ras-related nuclear protein (Ran) Guanosine-5′-triphosphate (RanGTP), then cleaved by Dicer, and finally turned into two single-stranded RNA (ssRNAs). The ssRNAs interact with RNA-induced silencing complex (RISC), protein complex [formed by Argonaute 2, Dicer, and transactivation response RNA binding protein (TRBP)]. The gene inhibition, mediated by miRNA-RISC complex, may take place through a site-specific cleavage, or by enhancing mRNA degradation or through translational inhibition (Cai *et al*., 2017; Treiber *et al*., 2019; Condrat *et al*., 2020; Matsuyama and Suzuki, 2020).

miRNAs are small non-coding RNAs, approximately containing 22–24 nucleotides, synthesized by enzymes called RNA polymerase II and III. In the nucleus, through out a maturation process the primary miRNAs are converted into miRNA precursor and then translocated to the cytoplasm where they mediate gene inhibition through miRNA-RISC complex (Fig. 1).

miRNAs regulate gene-expression post-transcriptionally by modulating mRNA degradation and altering protein levels. These processes are considered the primary molecular mechanism responsible for some pathological processes including cancer (Thomson *et al*., 2006). Remarkably, both innate and adaptive immune responses are affected by miRNAs, leading to their effects on the clinical symptoms of different diseases (Raisch *et al*., 2013; Cheng *et al*., 2014). For instance, miRNAs exert important functions in many aspects of the regulation of immune cell function by targeting inflammation-associated genes, including toll-like receptors (TLRs). Parasite recognition by TLRs leads to macrophage activation and control of *Leishmania* infection *via* the orchestrated production of pro-inflammatory and microbicidal effector molecules (Gallego *et al*., 2011). As a pathogenicity strategy, *Leishmania* parasites are able to change the TLR signalling pathways by modulating the expression level of miRNAs in infected-macrophages to subvert the host immune responses (Muxel *et al*., 2018*a*). Furthermore, miRNAs can also act as physiological ligands of specific TLRs and initiate the signalling cascade of immune responses (He *et al*., 2014; Bayraktar *et al*., 2019).

## miRNAs and diseases

**Fig. 2.**
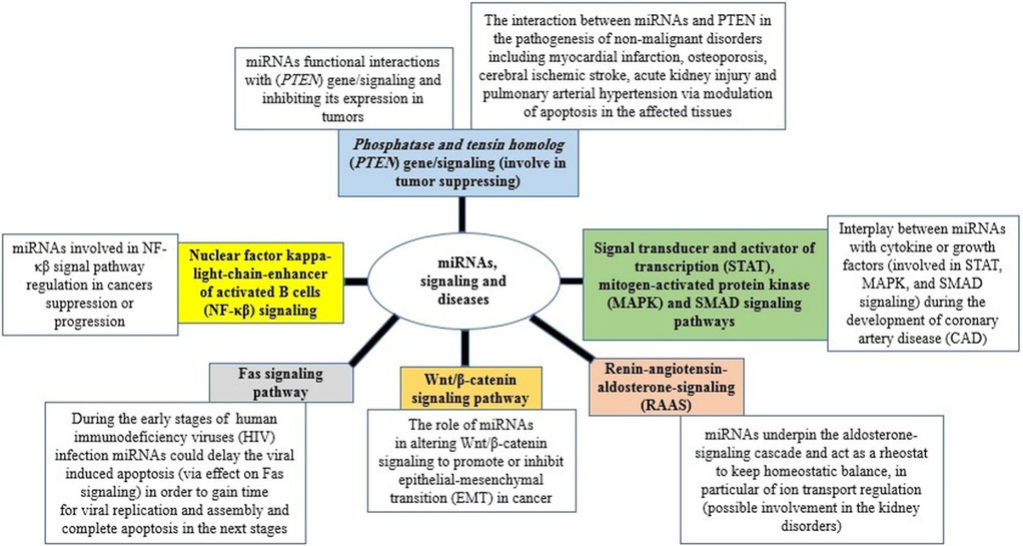
miRNAs in signalling pathways and diseases.

The ability of miRNAs to usurp different signalling pathways and consequently change the cellular response and the outcome of diseases is a hotspot in medical research science nowadays (Fig. 2) (Yang and Wang, 2016; Butterworth, 2018; Barbu *et al*., 2020; Gorabi *et al*., 2020; Lei *et al*., 2020; Ghafouri-Fard *et al*., 2021). Furthermore, miRNAs have been also suggested as valuable biomarkers in the treatment, diagnosis, and prognosis (Ali Syeda *et al*., 2020; Chakraborty *et al*., 2020; Chandan *et al*., 2020; Condrat *et al*., 2020; Matsuyama and Suzuki, 2020; Tribolet *et al*., 2020).

In this sense, many diseases have been associated with changes in the expression level of miRNAs, including systemic rheumatic diseases, nervous system disorders, sepsis, cardiovascular disease and different type of cancers such as breast, ovarian, cervical forms (Ceribelli *et al*., 2011; Abd-Aziz *et al*., 2020; Ali Syeda *et al*., 2020; Condrat *et al*., 2020). In addition, several investigations have recently demonstrated changes in circulating miRNAs in response to different infectious diseases, increasing the possibility for a new diagnostic tool (Acuña *et al*., 2020; Tribolet *et al*., 2020), even before the pathogen could be directly distinguished and prior to the onset of seroconversion (Stewart *et al*., 2013; Biswas *et al*., 2019). Thus, alterations in blood miRNA profiles have been associated with pathogens or pathologies such as Hendra virus (Stewart *et al*., 2013), tuberculosis (Zhang *et al*., 2013) and Ebola (Duy *et al*., 2016), human immunodeficiency virus (HIV) (Biswas *et al*., 2019) and malaria (Li *et al*., 2018), including differentiating complicated and uncomplicated *Plasmodium vivax* malaria (Kaur *et al*., 2018). Interestingly, miRNAs have also been highlighted in influenza infections (Scheller *et al*., 2019) and rhinoviruses (Hasegawa *et al*., 2018). Therefore, the potential diagnostics use of miRNAs with other respiratory viruses, such as the severe acute respiratory syndrome coronavirus 2 (SARS-CoV-2), is completely reasonable. Two recently works have reviewed the literature on the potential role of cellular miRNAs in the SARS-CoV-2-host interplay as a therapeutic option in coronavirus disease 2019 (COVID-19) patients (Fani *et al*., 2021; Zhang *et al*., 2021). The main conclusions of these two works are that miRNAs can inhibit the SARS-CoV-2 infection by interfering in various biological processes; blocking the angiotensin-converting enzyme 2 (ACE2) or the transmembrane protease serine 2 (TMPRSS2) as well as regulate the cytokine storm. Also, miRNAs-based therapeutics could be used in the nanovaccines.

## miRNAs during host−parasite interactions

Host−pathogen interactions lead to modifications in signalling and physiological processes in host cells that induce the miRNA-mediated post-transcriptional regulation of genes involved in different cellular mechanisms such as the inflammatory response during the induction of the immune response (innate and adaptive). Protozoan parasites including *Leishmania*, *Toxoplasma*, *Plasmodium* and *Trypanosoma* are able to change and affect host cell signalling and cellular mechanisms to their favour for developing pathogenicity in the infected host cells. The investigation of miRNAs as effective agents in regulating of such processes can help to understand more deeply the biology and pathogenicity of these parasites (Acuña *et al*., 2020; Paul *et al*., 2020).

It has been revealed that *Plasmodium* parasite up-regulates several host miRNAs that target proteins involved in immune response and down-regulates miRNAs that contribute to the inhibition of parasitic translation, host cell proliferation, metabolism and survival (Paroo *et al*., 2009; Lourembam *et al*., 2013). *Toxoplasma* parasite features its own miRNA processing system and is able to secret exosomes that contain miRNAs (Menard *et al*., 2019). The expression of miR-146a and/or miR-155 in infected host cells with *Leishmania*, *Toxoplasma* and *Plasmodium* parasites reveal common characteristics that are implicated in the subversion of the host immune response (Guerfali *et al*., 2008; Hentzschel *et al*., 2014; Frank *et al*., 2015; Acuña *et al*., 2020). By detecting the higher parasite burden in the liver and spleen of *Leishmania donovani*-infected miR-155 knockout mice, it was confirmed the effect of this miRNA on the host immune response in VL infection. *Leishmania* antigen-stimulated splenocytes from miR-155 knockout mice produced lower levels of T helper cell 1 (Th1)-associated interferon gamma (IFNγ) compared to controls (Varikuti *et al*., 2019). A broad view regarding the role of miRNAs in protozoan parasites infections and the interaction with host cells has been briefly reviewed in recent years (Acuña *et al*., 2020; Paul *et al*., 2020). In this sense, Table 1 summarized the critical role of miRNAs in some parasitic diseases and their pathological impact or clinical application.

**Table 1.** miRNAs in parasites and parasitic diseases and host-parasites interactions

Parasite or parasitic diseases	Expressed differential miRNAs	Pathological impact or clinical application	References
Chagas disease	Higher expression level miR-208a in plasma samples	TGF-*β* stimulation and regulation of genes involved in cardiac hypertrophy and fibrosis	Lacerda *et al*. (2018)
*Entamoeba histolytica*	Dysregulation of miRNAs in epithelial colon cells: Up-regulation of miR-526b-5p, miR-643, miR-615-5p, miR-525 and miR-150, and a down-regulation of miR-409-3p	Impact on the expression of genes involved in biosynthesis of unsaturated fatty acids, ubiquitin-mediated proteolysis, PI3K/AKT signalling pathway, mRNA surveillance pathways, and apoptosis	López-Rosas *et al*. (2019)
*Cryptosporidium parvum*	Down-regulation of miR-18b-3p, miR-34b-5p, miR-3591-3p and miR-3976 after infection	Regulation of both epithelial immune responses and apoptotic processes	Wang *et al*. (2019)
Cystic echinococcosis	egr-let-7 and egr-miR-71	For early diagnosis and monitoring in human plasma	Alizadeh *et al*. (2020)
Alveolar echinococcosis	miR-483-3p	For early diagnosis and monitoring in human plasma	Ren *et al*. (2019)
Schistosomiasis infection	Bantam, miR-2a-5p, miR-2c-3p and miR-3488 in sera from patients	Diagnosis and monitoring treatment effectiveness	Meningher *et al*. (2017)
	miR-21 and miR-96	Activate the SMAD signalling pathway to promote schistosomiasis-associated hepatic fibrosis	Chen *et al*. (2019)
	miR-351	Promotes hepatic fibrosis by targeting the vitamin D receptor (VDR)	
	miR-146a/b	Plays a protective role in hepatic schistosomiasis by regulating differentiation of macrophages into M2 cells	
	miR-203-3p	Inhibiting schistosomiasis-induced liver fibrosis	
	Let-7b	Inhibits liver fibrosis in schistosomiasis through multiple mechanisms	
	miR-182	Regulating the specialization of regulatory T cells	

In the current study, we have focused on the role of miRNAs expressed in *Leishmania* parasites and by their host cells that can explain the immunobiology of subversion, pathogenicity, survival, replication, drug resistance and treatment of these parasites.

## miRNAs expressed in *Leishmania* parasites

The identification and characterization of miRNAs in *Leishmania* parasites and their plausible biological functions can facilitate the discovery of potential therapeutic targets in leishmaniasis. Some computational strategies have suggested that the target genes of several miRNA-like elements expressed in *L. major* were related to the multidrug resistant protein such as adenosine triphosphate (ATP) binding cassette (ABC) transporter and also ribosomal protein, hydrolase and exonuclease and RNA binding proteins (Chandra Sahoo *et al*., 2013).

The antiproliferative and apoptotic effect of trans-dibenzalacetone (DBA, a synthetic monoketone analogue of curcumin) on *L. donovani* has been previously reported (Chauhan *et al*., 2018), and several miRNAs including hsa-miR-151a, hsa-miR-15b and hsa-miR-30c-1 were identified as down-regulated markers in DBA treated intracellular amastigotes in comparison with untreated parasites (Singh and Chauhan, 2018). On the other hand, miR-15b targets *B-cell lymphoma 2 (Bcl-2)* and the caspase signalling promoting apoptosis (Guo *et al*., 2009). Additionally, *autophagy-related protein 5 (ATG5)*, as a target gene of miR-15b, is required for ATG8 dependant autophagy and phospholipid balance in the mitochondrion in *L. major* (Williams *et al*., 2012).

miR-151a plays a role in the regulation of cellular respiration and ATP production by targeting cytochrome b. The downregulation of miR-151a, after DBA parasites treatment, induced mitochondrial dysfunction in *Leishmania* parasites (Zhou *et al*., 2015; Singh and Chauhan, 2018). miR-30a-3p is overexpressed in *Leishmania* infected cells (Singh *et al*., 2016), however, it is downregulated in DBA-treated parasites, suggesting that the down-regulation of this miRNA could inhibit the replication and virulence of *Leishmania* parasites (Singh and Chauhan, 2018).

The activity of *ATG4* (autophagy-related proteins) is required for parasite viability, and it has been identified as a target of miR-30c (Williams *et al*., 2009, 2013; Singh and Chauhan, 2018). The down-regulation of *ATG4* inhibits the cell viability of *Leishmania* parasites through the regulation of miR-30c expression. Summing up, DBA plays a major role in parasite survival and replication by affecting the expression of specific miRNAs which regulate the balance between autophagy and apoptosis (Singh and Chauhan, 2018).

## miRNAs expressed in *Leishmania*-infected host cells, tissues and sera

*Leishmania* parasites lead to the subversion/modulation of the innate immune response and cellular metabolic pathways in the host cells. Many host gene expression and signalling pathways are targeted by these parasites to modify host defences including immune activation, oxidative damage, antigen presentation and apoptosis, leading to parasite survival and replication. However, the molecular mechanisms used by these parasites to subvert the immune response are not fully clarified. Therefore, miRNA could be one of the most important regulatory factors to manipulate the host cells after infection (Diotallevi *et al*., 2018).

miRNAs play fundamental roles on macrophage activation, polarization, tissue infiltration and resolution of inflammation. They can balance between pro- and anti-inflammatory signalling, integrating stimulus from damage-associated molecular patterns (DAMPs), pathogen-associated molecular patterns (PAMPs) and inflammatory and anti-inflammatory cytokines such as transforming growth factor beta (TGF-*β*), IFN*γ*, glucocorticoids, interleukin 4 (IL*-*4) among others (Curtale *et al*., 2019). For instance, miR-155 expression is significantly enhanced when macrophages are polarized to the M1 phenotype; whereas it was considerably reduced in M2-polarized macrophages (Cai *et al*., 2012). miRNAs have been indicated as important players potentially participating in the modulation of the early phase as well as the resolution of inflammation (Curtale *et al*., 2019). Accordingly, miRNAs profiling in *Leishmania-*infected macrophages could reveal valuable information regarding immune responses, pathogenicity, survival, diagnosis, treatments and other biology aspects of these parasites.

### Expression patterns of miRNAs in peripheral blood mononuclear cells (PBMCs) and macrophages

The differential expression pattern of miRNAs as well as their relationship with the immune response and parasite load have been recently investigated in PBMCs and splenic leucocytes (SL) of Canine VL (CVL)-infected dogs by *L. infantum* (Bragato *et al*., 2018*a*, 2018*b*; Melo *et al*., 2019). In infected PBMCs, miR-21, miR-194, miR-424 and miR-451 showed a three-fold expression increase, miR-192, miR-371 and miR-503 denoted two-fold increase in their expression, whereas a two-fold decrease in miR expression level was detected for miR-150 and miR-574. The parasite load in PBMCs was correlated to the differentially expressed miRNAs, supporting the strong positive correlation with the expression of miR-194, a positive correlation with miR-371 expression, and a negative correlation with miR-150 expression in PBMCs (Bragato *et al*., 2018*b*). The increase level of miR-194 could be a mechanism to regulate the secretion of inflammatory cytokines, such as tumour necrosis factor alpha (TNF-*α*), modulating *Leishmania* parasite burden in infected animals (Bragato *et al*., 2018*b*). Interestingly, miR-194 also showed a strong positive correlation with serum urea of CVL infected dogs, suggesting that miR-194 could be useful as a possible early plasma biomarker in renal lesion of dogs infected with CVL (Wang *et al*., 2014; Esch *et al*., 2015). The expression of miR-371 was also increased in infected-PBMCs and showed a positive correlation with parasite load in PBMCs, suggesting that this miR could be associated with permissive immune response in CVL. Furthermore, miR-194 represented a potential negative correlation with haemoglobin concentration and miR-371 illustrated a strong negative correlation with erythrocyte globular volume (Bragato *et al*., 2018*b*). miR-150 as a detected down-regulated miRNA exhibited a negative correlation with *Leishmania* parasitic load in the blood (Zhou *et al*., 2007). miR-150 is probably acting in hypergammaglobulin and also in the development of regulatory B-cells, by enhancing the *Leishmania* parasite load due to T-cell suppression in CVL. On the other hand, the reduction in Natural Killer (NK) cells, modulated by miR-150, could be associated with the higher parasite burden in the PBMC of CVL-infected animals (Bragato *et al*., 2018*b*).

Similarly, microarray analyses indicated the enhanced expression level of miR-7, miR-21, miR-148a and miR-615, and the downregulation of miR-125a, miR-125b and miR-150 in infected-SL compared to control leucocytes. miR-148a targets genes involved in the regulation of apoptosis such as *FAS* and FAS *ligand* (*FASLG*) suggesting the role of this miRNA in the death of CD4^+^ and CD8^+^ cells in CVL-infected dogs (Melo *et al*., 2019). miR-615 targets *ligand-dependant nuclear corepressor (LCoR)*, a derepressor of peroxisome proliferator-activated receptor gamma (PPAR*γ*), which increases the phagocytic capacity of splenic macrophages (Jiang *et al*., 2011). miR-21 as another up-regulated miRNA probably contributes in the reduction of TNF-*α* level, which could lead to the increase of splenic parasite load and disease progression (Alves *et al*., 2009; Carissimi *et al*., 2014; Mazloom *et al*., 2016). As well known, IL-12 is an essential cytokine for activation of NK cells and IFN-*γ* production by T cells and polarization of immune response to Th1 during CVL (Strauss-Ayali *et al*., 2005). The transfection of infected-SL with a miR-21 inhibitor led to the increase of IL-12 cytokine and the T-box expressed in T cells (T*-*bet)/GATA-binding protein 3 (GATA*-*3) ratio (increasing Th1 profile population), and reduced *Leishmania* parasite load in infected-SL and revealed the interesting role of miR-21 in the inhibition of IL-12. These data highlighted that *L*. *infantum* infection changed the expression of miRNAs in *L. infantum* infected-PBMCs and -SL and that miRNAs including miR-21, miR-194, miR-371 and miR-150 interfered in the cellular immune response of *L*. *infantum*-infected dogs and also suggested such miRNAs as a plausible therapeutic target in CVL (Melo *et al*., 2019).

Evidences have shown that within the first 24 h of *L. major* infection of human primary macrophages induce a rapid change in the host miRNA profile. Alterations in the levels of miR-22, miR-133b, miR-155 and miR-210 have been associated with the host cell responses to apoptosis (Cheng *et al*., 2005; Lemaire *et al*., 2013). The expression level of miR-210 is significantly increased from 6 h to 24 h after *L. major* infection of macrophages. After silencing miR-210, the caspase-3 activity (as an apoptotic indicator) increased in HeLa cells (Cheng *et al*., 2005). Therefore, miR-210 up-regulation in *Leishmania*-infected macrophages might participate in the anti-apoptotic response of infected macrophages *via* caspase-3 inhibition.

In the same way, *L. major* infection induced the expression level of miR-24-3p as an anti-apoptotic factor in the first hours of infection in favour of its survival. miR-24-3p can interact and regulate *caspase 3* gene to expand the life time of macrophage and establish the parasite infection (Lasjerdi *et al*., 2020). Accordingly, the use of an antagomir-24-3p might be a possible therapeutic strategy for *L. major* treatment.

Additionally, the use of miR-15a mimic, miR-155 inhibitor or both of them increases the apoptosis rate of infected macrophages *in vitro*, and reduces the size of lesions *in vivo* within 6 weeks after the infection (Gholamrezaei *et al*., 2020) suggesting that miRNA-based therapy could be a possible novel treatment for cutaneous leishmaniasis.

The let-7 miRNA family is conserved from parasites to humans and correlated with the acute innate immune response, cell differentiation, development and therapeutic strategies by targeting *caspase-3* (Lee *et al*., 2005; Boyerinas *et al*., 2010). Let-7a is also able to induce cell apoptosis and cell cycle arrest (Zhao *et al*., 2018). The increased level of let-7a probably manipulate host cells in order to alter miRNA levels and regulate macrophage functions during infection (Hashemi *et al*., 2018*b*). Inhibiting let-7a by using a locked nucleic acid (LNA) oligonucleotide (Ørom *et al*., 2006) increased the apoptotic and necrotic process of *L. major-*infected human monocyte-derived macrophages *in-vitro* (Hashemi *et al*., 2018*a*). Since apoptosis suppression is a strategy used by *Leishmania* parasites to evade the host immune response (Gupta *et al*., 2016), the inhibition of let-7a might revealed new insights for the treatment of leishmaniasis.

Unfolded protein response (UPR) (endoplasmic reticulum (ER) stress response) is an evolutionary conserved mechanism aimed to restore ER homeostasis and ensure cell survival (Schröder, 2008). *L. infantum* is able to induce UPR as a critical pathway to promote infection progression in macrophages (Dias-Teixeira *et al*., 2016; Galluzzi *et al*., 2016). Different miRNAs have been shown to participate in UPR signalling (Maurel and Chevet, 2013). Thus, the UPR-activated transcription factor sXBP1 is able to up-regulate the expression of miR-346 in *L. infantum-* and *L. viannia-*infected macrophages (U937 and THP-1). For example, *RFX1*, a miR-346 predicted target gene, was significantly down-regulated 48 h post-infection. Additionally, several *major histocompatibility complex (MHC)*- or *interferon-associated* genes were suggested as targets of miR-346, indicating a critical role of this miRNA on regulating macrophage functions and as an attractive druggable anti-*Leishmania* drug target (Diotallevi *et al*., 2018).

TLR2 and TLR4 mediated *L. amazonensis* recognition and infectivity resistance in macrophages. In addition, myeloid differentiation primary response 88 (MYD88)-dependant receptors probably play a role in macrophage activation in response to *L. amazonensis* (Muxel *et al*., 2018*a*). It has been shown that the knockout of *TLR2*, *TLR4* and *MYD88* genes changed the rate of expressed miRNAs modulated in murine bone marrow-derived macrophages infected by *L. amazonensis*, including the down-regulation of let-7e expression, and then increased the parasites burden in these cells compared to the control. let-7e regulates pro- and anti-inflammatory responses during infection or TLR/PAMP stimulation by inducing NF-*κβ* (nuclear factor kappa-light-chain-enhancer of activated B cells) activation and cytokine production. Based on these results, the expression of miRNAs including let-7e, let-7f and let-7g requires MYD88, TLR2 and TLR4 signalling during *L. amazonensis* infection, highlighting the role of TLR pathway in the transcriptional and post-transcriptional regulation of gene expression during *Leishmania* infection (Muxel *et al*., 2018*a*). As abovementioned, TLR2, TLR4 and MYD88 exerted a regulatory function in miRNA expression, such as let-7e, during the course of infection. TLR2, TLR4 and MYD88 signalling changed the expression of genes involved in polyamine/nitric oxide (NO) production in *L. amazonensis*-infected macrophages. Let-7e affected *L. amazonensis* infectivity by regulating L-arginine metabolism. *Leishmania* parasites survival in macrophages depended on the deviation of L-arginine metabolism to the production of polyamines (Muxel *et al*., 2018*b*). Therefore, let-7e inhibition indirectly affected the expression of genes involved in L-arginine metabolism, increasing NO production and the subsequent parasite infectiveness (Muxel *et al*., 2018*a*). There are several studies that have highlighted the differential expression of miRNAs in *Leishmania*-infected macrophages (Table 2).

**Table 2.** Differential expression of miRNAs in *Leishmania*-infected macrophages

*Leishmania* spp.	Macrophage	miRNAs	Effect on the cell responses	References
*L. major*	Bone marrow-derived macrophages (BMDM)	miR-101c, miR-129 and miR-210	Suppressing autophagic response and increasing pathogenicity	Frank *et al*. (2015)
	THP-1	Up-regulation of miR-146a-3p and miR-146a-5p	Targeting TGF-*β* signalling pathway	Nimsarkar *et al*. (2020)
*L. infantum*	J774 macrophages	Up-regulation of miR-155	Suppressing immune response	Silva *et al*. (2018)
*L. donovani*	Human monocyte-derived macrophages (HsMDM) and THP-1	Up-regulation of miR-30a-3p	Suppressing autophagic response and increasing pathogenicity	Singh *et al*. (2016)
	THP-1	Has-miR (30, 93, 6-5p, 106, 155) and let-7c and 7f-5p	Regulation of TGF-*β* signalling pathway in post-kala-azar dermal leishmaniasis (PKDL)	Kumar *et al*. (2020a)
		miR-93, miR-143, miR-155, miR-221, miR-335 and let7c	Negative regulation of apoptosis process (restricting normal functions of macrophage activation in PKDL)	
		Up-regulation of hsa-miR-146, miR-9, miR-106, miR-155, miR-221 and miR-324	Reduction of IFN-*γ* signalling to favour disease progression during PKDL	
	RAW 264.7 mice macrophage	mir-328	Regulation of phagocytosis and phagocytic vesicle formation	Olivier *et al*. (2005), Degrossoli *et al*. (2011), Frank *et al*. (2015), Tiwari *et al*. (2017)
		miR-3473f, miR-763 and miR-8113	Negative regulation of apoptotic process (restricting normal functions of macrophage activation)	
		Up-regulation of miR-6996	Involved in lipophosphoglycan (LPG) and gp63 related signalling	
		Down-regulation of miR-3473f and miR-8113	Regulation of T cell proliferation, differentiation and Th1/Th2 dichotomy in pathogenicity of parasite	
		Overexpression of miR-6973a	IL-12 biosynthesis	
		Up-regulation of miR-3620	Regulation of cellular iron homoeostasis	
		miR-3620 and miR-6385	Regulation of hypoxia	

### The regulatory function of miRNAs on T cell subset in leishmaniasis

VL immunopathology is determined by mixed production of Th1/2 cytokines and the disease is fixed by an increased level of Th2 cytokine (Gupta *et al*., 2013). CD4^+^ T cells are main cell type responsible for the production of Th1/2 cytokine in the infected host cell by *Leishmania* parasites (Colpitts and Scott, 2010). During human VL, the plasticity of T cell proliferation and differentiation is related to the miRNA-mediated gene regulation which balance the Th1/Th2 or Th17/regulatory T cells (Tregs) type of immune response (Li *et al*., 2007; Nakahama *et al*., 2013). Th2 and Treg immune cells are critical in VL progression and Th1 and Th17 specific immune response are central to control this infectious disease. In this sense, miRNAs play important regulatory functions during the differentiation of naive CD4^+^ T and the balance among these specific skewed immune responses in *Leishmania* infection (Kumar *et al*., 2020*b*). Accordingly, some relevant information was summarized in Table 3 highlighting the regulatory function of miRNAs on T cell subset in leishmaniasis. Such data further show that miRNAs through a regulatory function to control CD4^+^ T cell differentiation, have a potential capacity to regulate immune signalling, cytokine production and immune cell migration to manage and control the human VL (Pandey *et al*., 2016).

**Table 3.** The regulatory function of miRNAs on T cell subset in leishmaniasis

VL		
miRNAs	Effect on immune responses	References
miR-29a, miR-29-b	Suppressing the Th1 specific protective immune response	Pandey *et al.* (2016)
miR-126 and miR-135	Suppressing the progression of Th2 type specific immune response	
let-7a-5p, miR-93 and miR-3622b-5p	Regulating Th17 and Treg cell differentiation and plasticity	
Up-regulation of miR-7a-1-3p, miR-690, miR-6994-5p, miR-574-5p and miR-7235-5p	Suppressing of transcription factors that were involved in the differentiation of naive CD4^+^ T cells into Th1 phenotype	Kumar *et al.* (2020*b*)
Down-regulation of miR-93-3p, let 7j, 486a-3p and miR-3473f	Targeting transcription factors responsible for the transformation of naive CD^+^ T cells to Th2 phenotype	
Up-regulation of miR-6994-5p and miR-5128	Targeting genes related to IFN-*γ* pathway	
Up-regulation of miR-7093-3p, miR-5128, miR-574-5p and miR-7235-3p	Targeting IL-12 receptor and may deregulate the IFN-*γ* mediated signalling	
Down-regulation of miR-340-5p	Targeting IL-4 and increasing the IL-4 production	
miR-155	Increasing CD4^+^ Th1 responses and IFN-*γ* production by targeting suppressor of cytokine signalling-1 (SOCS1) and Src homology-2 domain-containing inositol 5-phosphatase 1 (SHIP-1) leading to restriction of VL infection	Varikuti *et al.* (2019)
CL (*L. major* infection)		
miRNAs	Effect on immune responses	References
miR-10a	IL-12/IFN*γ*-influenced miR-10a controlled subsequent IFN*γ* production in Th1-Treg cells (regulating Th1-related Treg cells)	Kelada *et al.* (2013)
miR-182	IL-4-regulated miR-182 prevented IL-2 production in Th2-Treg cells (regulating Th2-related Treg cells)	

### miRNAs expressed in *Leishmania*-infected tissues and sera

Abnormal lipid profiles were reported in VL patients (Liberopoulos *et al*., 2014; Tsimihodimos *et al*., 2018), and had been observed in an animal VL infection model highlighting the fascinating association between the change of lipid metabolism (altered expression levels of lipid metabolic genes) and the liver miR-122 levels (Ghosh *et al*., 2013). miR-122 represents more than 70% of liver-miRNAs and is responsible for liver homoeostasis and lipid metabolism (fatty acid and cholesterol metabolism) (Elmen *et al*., 2008; Girard *et al*., 2008). RNase III endonuclease Dicer1 is able to process the change of pre-miRNAs to the mature form in the cytoplasm (Filipowicz *et al*., 2008). It has been indicated that leishmanial-metalloprotease glycoprotein 63 (gp63), a Zn-metalloprotease, targets Dicer1 inducing a decrease of miR-122 activity in human hepatic cells, as well as in *L. donovani*-infected mouse liver (Ghosh *et al*., 2013). This strategy also clarified the adaptation of parasites to combat regulatory RNA functions in host cells. Interestingly, the restoration of miR-122 or Dicer1 levels in VL mouse liver enhanced serum cholesterol and decreased liver parasite burden and survival (Ghosh *et al*., 2013). These results illustrated the strategies used by *Leishmania* parasites to control liver miR-122 and to modulate serum cholesterol.

The miRNA expression might change during pathological processes like localized cutaneous leishmaniasis (LCL) induced by *L. braziliensis*. It has been demonstrated that the expression of miR-193b and miR-671 are greatly associated with their target genes, *CD40 and TNF receptor (TNFR)*, underlining the critical function of these miRNAs in the expression of genes correlated to the inflammatory response in LCL. Interestingly, miR-193b and miR-671 correlate in patients who had faster wound healing (<59 days) but not in patients who need longer cure period (>60 days). Due to the association of such miRNAs with the control of inflammation and the healing time of LCL, they can be suggested as possible predictive markers of prognosis (Nunes *et al*., 2018).

The inflammasome, which is induced during *Leishmania* infection, involves the activation of caspase-1 and the release of the proinflammatory cytokines IL-1*β* and IL-18 that promote an inflammatory response and pyroptosis by triggering the release of more cytokines, the activation of other immune cells and programmed cell death (Di Virgilio, 2013). It was suggested that miRNAs exert a modulatory function in the assembly of the inflammasome complex (Nokoff and Rewers, 2013). The analysis of serum cytokines and the expression of circulating miRNAs in patients with CL showed increased levels of miR-7-5p, miR-133a, miR-146b, miR-223-3p and miR-328-3p, associated with the high levels of IL-1*β*, IL-6 and IL-17 compared to controls (Mendonça *et al*., 2020). These cytokine profiles in patients with CL may be triggering a Th17 immune response and enhancing IL-1*β* levels and inflammasomes activation. The overexpressed miRNAs profile in those patients is associate with the transcriptional control of several immune response genes, such as those involved in the regulation of programmed cell death (*DNAJB6, DNAJC5, IRS2, RBPJ, IGF1R, ECT2, MEF2C, FOXO3, FOXO1* and *TGFB2),* caspase activity (*NLRP3*, *SENP1*, *FOXL2*, *F3* and *SNCA*) and response to cytokine stimuli (*IRAK1*, *IL-6ST*, *TRAF6*, *MCL1* and *BCL2L1*). Data analysis showed an inverse correlation between the levels of IL-1*β* and the miR-7 and miR-223 in CL patients, whereas the levels of miR-133a, miR-146b and miR-328 showed positive values compared to IL-1*β* levels. These results indicated that miR-7, miR-133a and miR-223 played a critical role in the inflammasome activation (Mendonça *et al*., 2020). This information is very important to better understand the interplay between miRNAs and cytokines during CL infection.

The higher levels of serum exosomal miR-122 was recently identified as a good biomarker for liver diseases in leishmaniotic dogs. This result suggested that alterations of the lipid metabolism, low HDL (high-density lipoprotein) and high LDL (low-density lipoprotein) serum levels along with a lower miR-122 expression indicate a hepatic alteration induced by *L. infantum* in dogs (Loria *et al*., 2020). However, more investigations are needed to better define the role of miR-122 as a potential biomarker of hepatic damage/dysfunction during canine leishmaniasis.

## Differential expression of miRNAs associated with *Leishmania* survival, parasite burden, replication and infectivity

It has been revealed that *Leishmania* is able to reside successfully in the macrophages phagolysosomes, developing the parasitophorous vacuole (PV) that contains lysosomal markers including cathepsin D, lysosome associated membrane protein 1 (Lamp1) and Lamp2 (McConville *et al*., 2007). Accordingly, the Rab GTPases, involved in endosomal biogenesis, are considered potential targets of intracellular pathogens to subvert immune response (Spanò and Galán, 2018). *L. donovani* upregulates the expression of Rab5a (an early endosomal protein) in infected THP-1 macrophages by downregulating the expression level of miR-494. Subsequently, *Leishmania* parasites recruit and maintain Rab5a and early endosome associated antigen 1 (EEA1) on the PV allowing the parasites to reside in the early endosomal compartment without fusing with the lysosomes. The inhibition of the expression of *Rab5a* by promoting miR-494 expression or the knock down of *Rab5a* gene by siRNA will probably lead the internalized parasites endosome to the lysosomes fusion reducing the parasite survival and evasion (Verma *et al*., 2017). This information highlighted the essential role of miR-494 and *Rab5a*, for the survival of *Leishmania* parasites in human macrophages.

The analysis of miRNA profiling in *L*. *amazonensis*-infected macrophage showed that the lack of *L*. *amazonensis* arginase (*La*-*arg*^−^) led to distinct regulation of miRNA expression profiles in infected macrophages (Muxel *et al*., 2017). Seventy-eight percentage of altered miRNAs were upregulated in macrophages infected with *La*-WT parasites, whereas only 32% were up-regulated in macrophages infected with *La*-*arg*^−^. The lack of *L*. *amazonensis* arginase (*La-arg*^−^) inhibited the expression of two macrophage miRNAs, miR-294 and miR-721, which are involved in the interaction and regulation of *nitric oxide synthase 2 (NOS2)* and NO production. The absence of these miRNAs led to the reduction of parasite infectivity by promoting the NO production and suggesting *NOS2* as a target of the aforementioned miRNAs. *Leishmania* can use the parasite arginase/L-arginine metabolism to subvert NO production in macrophage, by inducing miR-294 and miR-721 (Muxel *et al*., 2017). Summarizing, these miRNAs could be pointed out as new targets for drug development.

Some strains of the *L. guyanensis* harbour a viral endosymbiont known as *Leishmania* RNA virus 1 (LRV1) (Ives *et al*., 2011) and TLR-3 recognition of these LRV1s increased *Leishmania* parasite burden and lesion swelling (Eren *et al*., 2016). However, the relationship between anti-viral innate immune responses and parasitic infection remains unknown. It seems that miR-155 is upregulated in macrophages infected with LRV1^+^ *L. guyanensis* in comparison with LRV1^−^ strain. The LRV1-driven miR-155 expression was dependant on TLR-3/TIR-domain-containing adaptor-inducing IFN-*β* (TRIF) signalling. This activation pathway increased parasite persistence by enhancing macrophage survival. Interestingly, phosphatidylinositol 3-kinase (PI3K)/protein kinase B (AKT) (PI3K/AKT) inhibition led to the reduction of LRV1-mediated macrophage survival as well as parasite burden. Moreover, miR-155-deficient mice significantly decrease the LRV1-induced disease severity and the Akt phosphorylation in macrophages obtained from the infected mice (Eren *et al*., 2016).

*L. donovani* led to the overexpression of miR-210 and hypoxia-inducible factor-1*α* (HIF-1*α*) in the host macrophages (Kumar *et al*., 2018) *via* a hypoxia-independent pathway (Chan *et al*., 2012; Singh *et al*., 2012). The miR-210 expression was transcriptionally controlled by *HIF-1α* and was dependant on *Leishmania*-induced *HIF-1α* activation (Lemaire *et al*., 2013; Kumar *et al*., 2018). Furthermore, macrophages infected with *L. donovani* and treated with siRNA for *HIF-1α* or antagomir-210 significantly reduced the parasitic burden and infectivity rate (Kumar *et al*., 2018). The upregulated miR-210 inhibited *TNF-α receptor* family leading to reduce the synthesis of different pro-inflammatory cytokines, which facilitated the parasite survival inside the macrophages. After silencing miR-210 with antagomir, pro-inflammatory cytokines genes such as *TNF-α* and *IL-12* were increased in miR-210 inhibited macrophages. This process also further promoted and increased the *Reactive Oxygen Species* (*ROS*) and NO production inducing the elimination of *Leishmania* parasites in infected macrophages (Kumar *et al*., 2018).

Interestingly, *Leishmania* infection was able to significantly up-regulate the expression level of host c-Myc inducing miRNA suppression. Indeed, c-Myc silencing decreased the intracellular survival of parasite suggesting that c-Myc is required for the pathogenicity of *Leishmania* (Colineau *et al*., 2018). Accordingly, c-Myc inhibitors can be considered as a possible therapeutic target for leishmaniasis (Whitfield *et al*., 2017).

Melatonin, the darkness-signalling hormone, plays a critical role in the modulation of macrophage activation and controlling the inflammatory response during parasitic infection (Markus *et al*., 2018; Xia *et al*., 2019). Recently, exogenous melatonin treatment of BALB/c macrophages was found to decrease *L. amazonensis* parasite burden and modulated host miRNAs expression profile (miR-294-3p, miR-302d-3p and miR-30e-5p) (Fernandes *et al*., 2019). Melatonin treatment also decreased IL-6, monocyte chemoattractant protein*-*1 (MCP-1), RANTES (Regulated upon Activation, Normal T Cell Expressed and Presumably Secreted) and macrophage inflammatory protein-2 (MIP-2), as well as IL-10 levels in infected macrophages (Lebovic *et al*., 2001; Marçola *et al*., 2013; Fernandes *et al*., 2019). miR-294-3p targets *NOS2* mRNA decreasing *NOS2* expression and promoting infectivity (Muxel *et al*., 2017) and its inhibition drives high expressions of *TNF* and *Mcp-1/chemokine ligand 2 (Ccl2)* that reduce infectivity. In addition, miR-302d has also been described as a regulator of *NOS2* expression (Farlik *et al*., 2010; Smith *et al*., 2017), and melatonin treatment or miR-302d-3p or miR-30e-5p inhibition enhanced *NOS2* mRNA expression and NO production, decreasing macrophages infection. In fact, melatonin treatment of *Leishmania*-infected macrophages changes the balance of L-arginine metabolism by inducing NOS2 in detriment of arginase 1 (Arg1) and thus altering infectivity (Fernandes *et al*., 2019).

**Fig. 3.**
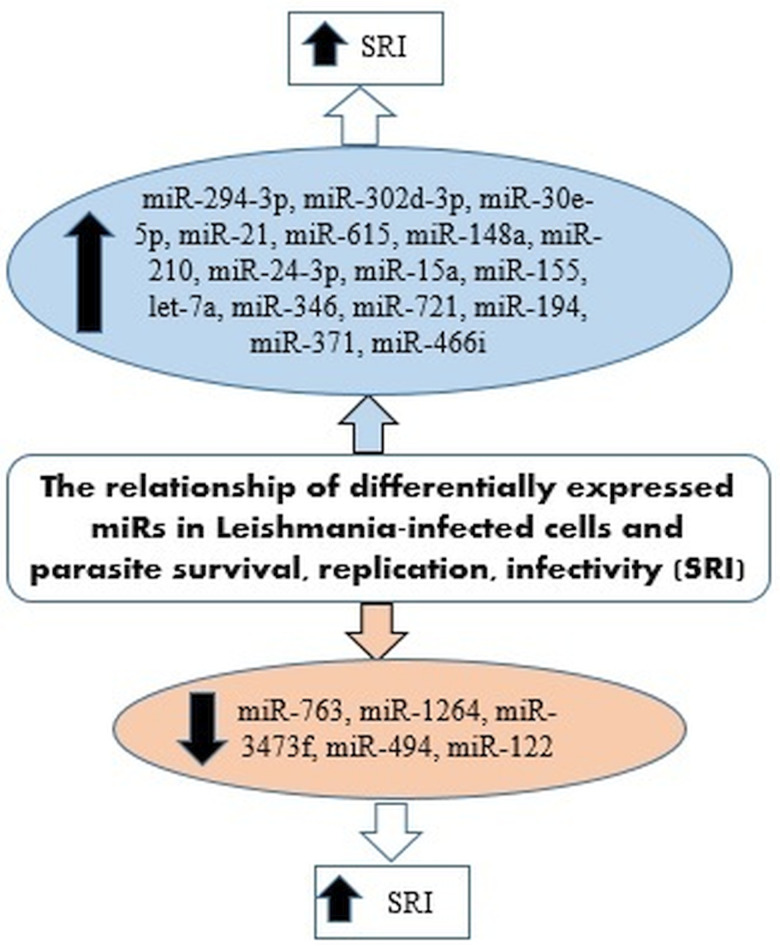
miRNAs expressed in *Leishmania*-infected host cells involved in the survival, replication and infectivity of the parasite.

IL-12 produced by dendritic cells (DCs) is essential for starting a host protective Th1 cell response, but miR-21 has been indicated as a key negative regulatory factor of the expression of *IL-12* mRNA during leishmaniasis infection. High levels of miR-21 were associated with low expressions of *IL-12* mRNA in DCs infected with virulent *Leishmania* strains. Furthermore, silencing miR-21 enhances the *IL-12* expression in DCs, during the infection with a virulent strain. These results suggest the critical role of miR-21 in mediating suppression of this cytokine. The infection of DCs with attenuated strains of *Leishmania* and suggesting that the levels of miR-21 could be measured as anti-leishmanial response of vaccines. Accordingly, lower levels of miR-21 could represent better immunogenicity and protective immune response of the vaccine (Bhattacharya *et al*., 2017; Gannavaram *et al*., 2019). Figure 3 has summarized several miRNAs expressed in *Leishmania*-infected host cells that could be involved in the survival, replication and infectivity of *Leishmania* parasites.

## Expression of miRNAs in host cells and parasite drug resistance

miRNAs play an important role in drug resistance by altering the drug transporters, receptors and ion channels, thus, reducing the sensitivity of drugs (To, 2013; Ren *et al*., 2015; Nawaz *et al*., 2019). The identification of miRNAs related to *Leishmania* parasites drug resistant could provide mechanistic details to combat drug resistance in leishmaniasis. Some studies revealed that *Leishmania* parasites induced the up-regulation of ABC transporters in macrophages by down regulating miR-763, miR-1264 and miR-3473f generating the efflux out of drugs (Singh *et al*., 2014; Tiwari *et al*., 2017).

Infections of mammals with *L. donovani* resistant (LD^R^) lead to aggressive pathologies as compared to their sensitive strains (LD^S^) coupled with higher levels of IL-10 and TGF-*β*. The IL-10 increases the upregulation of multidrug- resistant protein-1 which produces the efflux of antimonials drugs from LD^R^ infected-host cells (a key mechanism of antimony resistance) (Guha *et al*., 2014). Considering that miRNAs are involved in the control of cytokines expression (Garavelli *et al*., 2018), the alteration of miRNA profile in the host cell could be an effective strategy to ensure infection or drug resistance by pathogens. Accordingly, targeting miRNA pathway might be a novel strategy to control infection caused by pathogens such as LD^R^ parasites (Mukherjee *et al*., 2020). The clinical manifestations of *L. donovani* infection are related to the critical balance of pro- and anti-inflammatory cytokines which is obtained through the miRNA-mediated regulation and by targeting the miRNA modulators, HuR and protein phosphatase 2A (PP2A) (Goswami *et al*., 2020). Argonaute 2 (Ago2) phosphorylation may impair the binding of the protein with miRNAs and to the corresponding target mRNAs, therefore, the dephosphorylated form of Ago2 is required for miRNA activity (Chakrabarty and Bhattacharyya, 2017). On the other hand, phosphorylation and de-phosphorylation of Ago2 is controlled by PP2A and HuR. HuR is a miRNA derepressor protein and a miRNA sponge for specific miRNAs to negate their action on target mRNAs. HuR acts as a balancing factor of immune responses to disrupt the macrophage infection by the protozoan parasite. *Leishmania* parasites target HuR to promote the initiation of anti-inflammatory responses in infected macrophages. These parasites also induce the overexpression of PP2A that maintain Ago2 in dephosphorylated form, causing strong repression on the miRNA-targeted pro-inflammatory cytokines to promote an anti-inflammatory response in infected macrophages. HuR has an inhibitory effect on PP2A expression, and evidence suggested antagonistic miRNA-modulatory functions of HuR and PP2A which mutually balances immune response in macrophage by targeting miRNA function. Consequently, the expression of HuR and the simultaneous inhibition of PP2A can induce strong pro-inflammatory responses in the host macrophage to prevent the virulent antimonial drug sensitive or drug-resistant form of *L. donovani* infection (Goswami *et al*., 2020). LD^S^ and LD^R^ upregulate PP2A and downregulate HuR at various levels inducing different levels from anti-inflammatory to proinflammatory cytokine production and generating disease manifestations in the host. HuR expression alone is sufficient to remove LD^S^ infection, however, simultaneous increasing levels of HuR and inhibition of PP2A are needed to inhibit LD^R^ mediated infection (Mukherjee *et al*., 2020). Moreover, the analysis of predicted miRNAs with related binding sites in host cytokine transcripts identified a maximum number of interactions with IFN-*γ* transcript suggesting a possible and unknown function of IFN-*γ* in LD^R^ infection. Among other Th1 cytokines IFN-*α*, IFN-*β*, IL-12, TNF-*α* and IL-6 also represented significant amount of interactions. The major Th2 cytokines with probable miRNA binding sites contain IL-10 and TGF-*β* while Th-17 cytokines like IL-17 and IL-27 also showed considerable number of potential interaction sites. Among the identified miRNAs, miR-487b, miR-669d, miR-669a-5p, miR-1251, miR-1381-1 and miR-2139 showed minimum number of interactions (Mukherjee *et al*., 2020).

Antimony-resistant *L. donovani* (Sb^R^LD) parasites interact with TLR2/TLR6 to induce IL-10 by exploiting p50/c-Rel subunits of NF-kB in infected macrophages (Mukherjee *et al*., 2013). Most of the TLRs can exploit the universal adaptor protein MYD88 to activate the transcription factor NF-*κβ* (Jefferies *et al*., 2001). It has been indicated that infections of macrophages from *MYD88^−/−^* mice with Sb^R^LD significantly enhance the intracellular *Leishmania* parasite number coupled with the increased IL-10/IL-12 ratio in the culture supernatant in comparison with infections of wild type (WT) macrophages. In contrast, the infection with Sb^S^LD cannot induce such a process. Infections of *MYD88^−/−^* macrophages or *IL-12^−/−^* macrophages with Sb^R^LD induced high levels of IL-10 at 4 h, whereas the level of the same cytokine was increased after 12 h in WT macrophages, indicating that the absence of IL-12 favoured early binding of NF-*κβ* subunits to the *IL-10* promoter, leading to the increase of IL-10 levels. MYD88 signalling is critical in maintaining IL-12 levels, but the up-regulation of miR-466i after Sb^R^LD infection lead to the degradation of MYD88 and subsequently a reduction in IL-12 levels (Mukherjee *et al*., 2014, 2015). Consequently, the reduced levels of IL-12 activate *IL-10* promoter resulting an IL-10 increase in the host. Therefore, Sb^R^LD use a significant strategy to evade host anti-leishmanial immune responses by manipulating host MYD88 to its favour (Mukherjee *et al*., 2015). Thus, the selection of approaches to restore MYD88 signalling by targeting miR-466i might be an attractive tool in managing Sb^R^LD parasite-mediated leishmaniasis.

## Conclusions and future directions

The identification of parasite miRNAs and those induced in the host cell by the infection brought new insights and understanding regarding the pathogenesis and druggable targets against parasitic diseases such as *Leishmania* infections. For instance, high levels of miRNAs in a specific tissue or serum of infected animals suggested them as possible biomarkers for that disease. Despite of standardized protocols for the current clinical practise, miRNAs screening constitutes a reliable tool for future use. Further investigations will bring more criteria needed to be used as appropriate biomarkers, including accessibility, high specificity and sensitivity (Condrat *et al*., 2020). Moreover, this review intends to put together, most relevant information regarding *Leishmania*-specific miRNAs and their targets in hosts cells, as well as the mechanisms used by miRNAs to interfere with host pathophysiology of leishmaniasis at the molecular level. The investigation of exosomes and their miRNA contents will be very helpful for future chemotherapies and vaccination. These studies tried to identify, unique or highly different miRNA molecules as possible druggable targets. The transport and delivery of miRNAs by using exosomes is getting higher attention in parasitology and immunology fields due their capacity to modulate the host immune response. Although, the presence of miRNAs in parasitic exosomes has been largely investigated in helminth infections, it is a promising strategy in protozoan parasites (Nawaz *et al*., 2019). The design of specific inhibitors against those key miRNAs involved in protozoan parasites infection will facilitate the control of leishmaniasis and other infection diseases. In conclusion, the biological information related to miRNAs, parasite infection and the interplay with the host cells and immune response will illuminate future biomedical research. Since miRNAs have a great potential to lead a new class of theranostic tools, the identification of more specific miRNAs with highly specialized functions might provide novel guidelines for the management of parasitic diseases (Paul *et al*., 2020).
